# The effects of personality traits on entrepreneurial intention and creativity among Chinese and African college students in era of industry 4.0/5.0

**DOI:** 10.3389/fpsyt.2023.1098148

**Published:** 2023-01-19

**Authors:** Miaoxin Xu

**Affiliations:** Shanghai Jiao Tong University, Shanghai, China

**Keywords:** conscientiousness, openness, boundaryless mindset, entrepreneurial intention, creativity

## Abstract

Using samples of Chinese and African college students, this manuscript explored the effect of personality traits on entrepreneurial intention and creativity from the boundaryless career perspective. The empirical results showed that both Chinese and African college students’ conscientiousness and openness had significantly positive effects on entrepreneurial intention and creativity, respectively, and boundaryless mindset had a mediating effect in the relationship between conscientiousness, openness, entrepreneurial intention, and creativity. However, the moderating effects of GPA were quite different. While Chinese college students’ GPA strengthened the positive effect of boundaryless mindset on entrepreneurial intention and creativity respectively, African college students’ GPA weakened the positive effect of boundaryless mindset on entrepreneurial intention and had no significant moderating effect on the relationship between boundaryless mindset and creativity. This study was based on the empirical research of Chinese and African college students through a combination of contrastive and advance study methodology. It has provided new perspectives for exploring influencing factors and effects of employment performance in the context of Industry 4.0/5.0 and it has made theoretical and practical contributions to promote creativity and entrepreneurial intention.

## Introduction

Tracing back modern history, every industrial revolution was closely connected to innovative experts that came from higher education. Industrial revolution requires ground-breaking technology being invented and utilized by great experts, and higher education is the incubator of these kinds of talents. Since intelligent manufacturing, information, biotechnology, materials, and other technology have been booming in the 21st century, a new round of industrial revolution has now lead us into the era of Industry 4.0/5.0. Industry 4.0/5.0 is characterized by digitalization, networking, and intelligence, and these characteristics are mainly based on high level scientific technology. Since innovation and entrepreneurship are important methods of producing high level scientific technology (1, 2), many counties proposed innovation and entrepreneurship as important approaches to help business and organizations to cope with the new era. China proposed the concept of “mass entrepreneurship and innovation” in order to maintain its fast-developing momentum in Industry 4.0/5.0 and this strategy has gained increasing recognition, especially by developing countries. The characteristics of Industry 4.0/5.0 require employees to break free from mechanical and labor-intensive working style and to become more creative, meaning to propose novel and useful ideas (3) that range from creating new products to even new companies. College students are important sources for employee recruitment, and they also are one of the most creative groups of people with entrepreneurship potential. The concept of creativity and entrepreneurship are increasingly closely related in the field of higher education (4). As the driving factor, entrepreneurial intention is an important prerequisite and predictor for individuals to exhibit entrepreneurial behaviors in the future (5), so it is an important entry point for studying entrepreneurial behaviors. In parallel, creativity is the basis for organizations to perform innovative activities, like developing technology, providing services, creating products and procedures (6, 7). Creative individuals are the potential contributors for enterprise innovation and entrepreneurship; therefore, implementing appropriate methods to increase college students’ creativity and entrepreneurial intention can foster talents for the society and Industry 4.0/5.0. Industry 4.0/5.0 has inevitably changed the job market and decreased the demand of traditional labor force while the number of college graduates has been increasing (8). Therefore, it is significant to explore the promoting factors and the effects on college students’ entrepreneurial intention and creativity to prepare students with more skills and capabilities to cope with the employment uncertainty. This will also assist businesses with recruitment and predicting future work performance, possibly providing academics with new perspectives and methods to explore factors that influence work performance.

Personality traits dominate individual attitudes and behaviors, especially individuals’ work performance and mental health, and many personality traits are formed in adolescence (9). Once formed, these traits are relatively stable from changing. Therefore, we believe college students’ personality traits may have a great influence over their entrepreneurial intention and creativity. Because of its high predictability and applicability, the Big Five personality model is considered as an important method to study personality structure by many scholars at home and abroad. Previous studies have pointed out two dimensions in the Big Five personality model, conscientiousness and openness, are more closely related to entrepreneurial intention (10) and creativity (11). Therefore, this study was mainly focused on the effect of conscientiousness and openness on college students’ entrepreneurial intention and creativity. In the meanwhile, existing studies have pointed out that the Big Five personality traits affect employees’ professional interests and career attitudes (12). As Industry 4.0/5.0 continues to evolve, the platform for individual career development is broader and employment opportunities are more diversified. As a new career attitude, boundaryless mindset has attracted increasing attention from researchers and managers. This study will explore how conscientiousness, openness, and other personality traits affect college students’ boundaryless mindset and then further affect entrepreneurial intention and creativity.

In addition, this study focused on the different effects of economic and cultural factors on the relationship between variables. For example, under the influence of Industry 4.0/5.0, whether college students in countries with large economic and cultural differences will be affected differently? A comparative study can help us to better understand how personality traits directly affect college students’ boundaryless mindset and further affect their entrepreneurial intention and creativity. Therefore, this study took Chinese and African college students as research objects, and it is of great practical significance to compare and analyze the influence of personality traits and boundaryless mindset on entrepreneurial intention and creativity.

## Literature review and hypothesis development

### Conscientiousness, openness personality, entrepreneurial intention, and creativity

The Big Five personality model includes five main personality traits: extraversion, agreeableness, emotional stability, conscientiousness, and openness (13). Among them, conscientiousness includes two main connotations: achievement orientation and responsibility (14). Specifically, individuals with high conscientiousness yearn for achievement that they have a strong sense of purpose and willpower, hold responsibility, work earnestly, exhibit self-control, and strive to achieve their goals and aspirations (14). Openness describes individuals’ imagination, curiosity, sensitivity to esthetics, independent thinking and the acceptance level of novel ideas, new experiences, and non-traditional views. Openness makes a clear distinction of individuals who are willing to accept changes and new experiences and break routines, from individuals who are comfortable with the *status quo*, adhere to traditions, stick to routines, and follow rules (15). Individuals with high openness are more likely to accept different feelings, ideas, and opinions, adapt to the changing environment, challenge the *status quo*, and be good at thinking and proposing new problem solutions. On the contrary, individuals with low openness are more conservative that they prefer a familiar environment and tend to accept traditional ideas.

Entrepreneurial intention represents an individual’s desire and behavior preference for independent entrepreneurship in the future (16). Individuals with high conscientiousness tend to pay more attention to self-control (17) and usually maintain high independence. Through meta-analysis, Barrick and Mount (18) concluded that individuals with high conscientiousness have high achievement orientation, which is often manifested as ambitiousness and perseverance. These high conscientiousness traits are considered to be the core traits of entrepreneurs (19), in line with entrepreneurial aspirations and preferences. We therefore propose:

Hypothesis 1a: Conscientiousness is positively related to entrepreneurial intention.

Creativity refers to an individual’s ability to generate new and useful ideas Zhou and George (6). In a workplace, ideas must be suitable to solve problems to be innovative and implemented (20). A few studies on the relationship between conscientiousness and creativity have inconsistent results. Studies have found that the direct relationship between conscientiousness and creative performance is not significant (21); other studies have found that high conscientious individuals show low levels of creative behaviors in certain situations (22). If subordinates are closely monitored by supervisors or lack support from colleagues, high conscientious subordinates tend to comply with the expectations of the supervisor or behave consistently with colleagues, which will reduce individual creative behaviors (22). Some studies have pointed out that high conscientiousness may result in great creative performance even when individual creativity is not very strong (23). Other studies indicated that high conscientiousness not only strengthen the positive effects of work engagement on job performance, but also promotes the impact of work engagement on proactive learning (24). For college students, proactive learning is particularly important. Proactive learning is closely related to creativity, that individuals with this trait often can put forward new ideas. Therefore, we propose:

Hypothesis 1b: Conscientiousness is positively related to creativity.

By using the meta-analysis method, Brandstatter found that openness is significantly ’san related to entrepreneurial intention and entrepreneurial performance (25). Individuals with high openness tend to have three features critical among successful entrepreneurs: strong curiosity, rich imagination, and high intelligence (26). Highly open individuals are curious, more willing to accept new ideas, and often good at capturing new resources and opportunities. Therefore, we propose:

Hypothesis 2a: Openness is positively related to entrepreneurial intention.

Openness is an important factor in predicting creativity. Creativity originates from curiosity and imagination, which are the core of open personality traits. Individuals with high openness are often not confined to tradition (27), and are more willing to put forward and accept novel ideas and opinions that are not yet tested or seemingly unrealistic (28). Therefore, we propose:

Hypothesis 2b: Openness is positively related to creativity.

### Boundaryless mindset, entrepreneurial intention, and creativity

The concept of borderless career was first proposed by Defilippi and Arthur (29), and was defined as a series of work opportunities not limited to a single employment environment. Arthur and Rousseau pointed out that boundaryless career is the pursuit of independence and autonomy from the traditional organizational career arrangement, rather than completely relying on the career arrangement proposed by organizations (30). By reviewing the development of the concept of boundaryless career, Sullivan and Arthur proposed that boundaryless career should include two aspects: physical flow and psychological flow (31). Based on Sullivan and Arthur’s point of view, Briscoe et al. divided the psychological flow into boundaryless mindset and organizational mobility preferences, and they developed the corresponding scales. Boundaryless mindset refers to an individual’s psychological preferences and the ability to proactively pursue working relationships across boundaries (32, 33). Organizational mobility preference refers to an individual’s psychological tendency to cross the boundary of “real” and “physical” work flow (32). Since the research objects of this paper are ungraduated college students, most of whom have not yet been involved in a working department or organization, we mainly focus on the boundaryless mindset of college students. Drawing on the views of Briscoe et al. (32), we believed that if college students have the psychological preference and ability to proactively pursue learning and working relationships across professional boundaries, then they would have the tendency, highlighted by the boundaryless mindset, to cooperate and communicate across boundaries.

College students with boundaryless mindset like to pursue cross-disciplinary learning, interact and cooperate with people from different specialties, and seek learning opportunities outside their own specialties, so they can be closely connected, and this “small-circle” network helps to make sense of cross-disciplinary knowledge, thereby further increases entrepreneurial intention (34) and creativity (35).

The social network formed by people with high boundaryless mindset increases the sources and channels of an individual’s access to information. These individuals can identify and capture more opportunities, enhancing their entrepreneurial intention. For example, college students can increase the probability of finding entrepreneurial opportunities by participating in entrepreneurial training courses, entrepreneurial salons, and road shows. At the same time, learning more about the risk and profit involved in entrepreneurship from other entrepreneurs affects individual’s awareness of entrepreneurship. When individuals learn more methods to control risk and increase profit, they tend to be more inclusive of the risk of entrepreneurship, thus more willing to start a business. Individuals with boundaryless mindset also like to make acquaintances widely, including cultivating relationships with potential investors. The formation of these social networks helps to improve the ability to obtain entrepreneurial resources. When college students have both entrepreneurial opportunities and entrepreneurial resource as their support, entrepreneurial intention will increase. Therefore, we propose:

Hypothesis 3: Boundaryless mindset is positively related to entrepreneurial intention.

The social network formed by high boundaryless mindset also encourages individuals to acquire more information and knowledge. Cross-disciplinary learning and interaction with students of different specialties can stimulate students to put forward unique ideas in learning and extracurricular activities. Individuals with boundaryless mindset have strong willingness for cross-border communication and cooperation. While actively building the network to share their own knowledge, they also absorb the heterogeneous knowledge from others, and building on this, they integrate and apply knowledge, generating more new ideas (36). We therefore propose:

Hypothesis 4: Boundaryless mindset is positively related to creativity.

### The mediating effect of boundaryless mindset

Studies have shown that personality traits significantly affect individual career attitudes and career choices (32, 37). High conscientious individuals have a clear understanding of their role expectations. Such individuals with high self-control are sensitive to various social cues, and are more likely to seek new information and partner across borders (38). At the same time, individuals with high conscientiousness are reliable, which is not only reflected in being responsible for themselves, but also in being responsible for others and collectives. Sullivan and Arthur found that collectivism-oriented individuals are more likely to be aware of the opportunities for career psychological mobility and perceive their ability to move (31). Therefore, we believe that if college students are highly conscientious, they have relatively strong psychological preferences for cross-border learning and cooperation, and have the characteristics of boundaryless mindset.

Highly open individuals have strong curiosity, like to try new things and experiences (28). This curiosity is an important career adaptability (39), and Sullivan and Arthur believed that individuals with strong professional competence may experience more psychological flows (31). While low-openness individuals prefer a familiar environment, high-openness individuals are less comfortable with the *status quo* and prefer to explore unfamiliar environment to obtain new experiences and ideas (40). Therefore, high-openness individuals tend to learn and communicate with others across borders.

Combined with the above arguments, we propose:

Hypothesis 5a: Boundaryless mindset plays a mediating role between conscientiousness and entrepreneurial intention.

Hypothesis 5b: Boundaryless mindset plays a mediating role between conscientiousness and creativity.

Hypothesis 6a: Boundaryless mindset plays a mediating role between openness and entrepreneurial intention.

Hypothesis 6b: Boundaryless mindset plays a mediating role between openness and creativity.

### The moderating effect of GPA

Grade point average (GPA) reflects learning effectiveness and the comprehensive learning ability of a college student, including the ability to understand, absorb, and apply knowledge. Students with higher GPA usually have a larger knowledge base, higher learning capacity and better ability to capture information and opportunities. If their GPA is high, individuals who tend to learn and communicate with others across borders may obtain more opportunities, information, and knowledge, be encouraged to have entrepreneurial intention, and generate new ideas. We therefore propose:

Hypothesis 7a: GPA positively moderates the relationship between boundaryless mindset and entrepreneurial intention, that is, the higher the GPA, the greater the positive effect of boundaryless mindset on entrepreneurial intention.

Hypothesis 7b: GPA positively moderates the relationship between boundaryless mindset and creativity, that is, the higher GPA, the greater the positive effect of boundaryless mindset on creativity.

**FIGURE 1:**
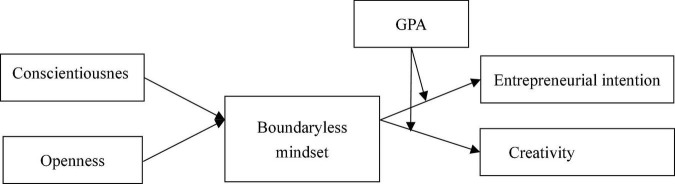
Conceptual model.

The model of this study is shown in Figure 1.

## Materials and methods

### Sample and data collection

This study chose Chinese and African college students as research objects, and the data was collected from one college in Shanghai and three universities in the Democratic Republic of Congo. The general quality of higher education in Shanghai is one of the highest in China, and Shanghai is a major gathering place for entrepreneurs. The Democratic Republic of Congo is the second largest country in Africa with the fourth largest population. Questionnaires were completed and collected electronically. After eliminating invalid questionnaires, we obtained 323 valid responses from Chinese college students and 238 valid responses from African. In the Chinese sample, males were accounted for 74.9%, females were 25.1%; 14.2% of the Chinese sample had received career development guidance in school, and 9% had taken entrepreneurship education courses in school. In the sample of African college students, males were accounted for 58.0%, females were 42.0%; 71.0% of the African sample had received career development guidance in school, and 56.3% had taken entrepreneurship education courses in school.

### Measures

The scales used in this study are all mature scales, and Likert’s five-point score was adopted.

The scales of conscientiousness and openness were referred to Saucier’s Big Five Personality Scale (41), which included six items. Exemplary items used for consciousness and openness were “solid” and “imaginative,” respectively. The reliability coefficient of the conscientiousness scale was 0.832 in Chinese sample data and 0.706 in African. The reliability coefficient of the openness scale was 0.765 in Chinese sample data and 0.784 in African.

The scale of boundaryless mindset was referred to the eight-item scale developed by Briscoe et al. (32), and was modified according to the characteristics of college students, for example: “I am enjoy working with people from different specialties.” The reliability coefficient of the scale was 0.873 in Chinese sample data and 0.910 in African.

The scale of entrepreneurial intention was adopted from the six-item scale developed by Westhead and Solesvik (42), for example: “My career goal is to become an entrepreneur.” The reliability coefficient of the scale was 0.910 in Chinese sample data and 0.891 in African.

The scale of creativity was referred to the four-item scale developed by Baer and Oldham (43), and was modified according to the characteristics of college students, for example: “I always take the lead in trying new ideas or new methods in front of my classmates.” The reliability coefficient of the scale was 0.863 in Chinese sample data and 0.915 in African.

The control variables in this study were gender, whether the individual received school career development guidance, and whether they took entrepreneurship education courses.

## Results

### Common method variance test

The data of all variables in this study were collected from the same subject. Homologous variance is difficult to avoid. Therefore, the Harman’s single factor test was used to investigate the homology deviation of the data. We used SPSS25.0 for computation, and seven factors were precipitated in Chinese and African sample data by principal component analysis without rotation. In Chinese and African samples, 67.767 and 71.514% of the total variance were explained, and the first factor explained 27.293 and 36.988%, respectively. The first factors were lower than 50% of the judgment standard (44), indicating the problem of homology deviation of each sample data was not serious.

### Confirmatory factor analysis

In this study, the confirmatory factor analysis (CFA) method was used to test the suitability of the overall research model. The CFA results after processing were shown in Table 1. The overall fitting indexes of the model in each sample data were within the acceptable range, indicating that the model in this study was well-adapted.

**TABLE 1:** Confirmatory factor analysis results.

Sample source	χ^2^	Df	χ^2^/Df	RMSEA	CFI	TLI	GFI
China	204.765	94	2.178	0.060	0.960	0.949	0.932
Africa	249.098	94	2.650	0.083	0.942	0.926	0.885

### Hypothesis testing

The mean, standard deviation, and correlation coefficient of the main variables in this study are shown in Table 2.

**TABLE 2:** Mean, standard deviation, and correlation of variables.

Variables	Sample	Mean	S.D.	1	2	3	4	5	6	7	8
1. Gender	A	0.749	0.434								
	B	0.580	0.495								
2. Career development guidance	A	0.142	0.350	0.011							
	B	0.710	0.455	-0.056							
3. Entrepreneurship education curriculum	A	0.090	0.286	-0.043	0.120*						
	B	0.563	0.497	0.125	0.371**						
4. Conscientiousness	A	6.313	1.272	0.007	0.024	-0.013					
	B	6.504	1.376	0.187**	0.103	0.275**					
5. Openness	A	6.326	1.057	0.134*	0.162**	-0.017	0.335**				
	B	6.180	1.457	0.140*	0.083	0.263**	0.637**				
6. Boundaryless mindset	A	4.161	0.589	-0.019	0.131*	0.054	0.306**	0.301**			
	B	3.879	0.830	0.164*	-0.015	0.069	0.447**	0.458**			
7. GPA	A	3.393	1.099	0.084	-0.033	0.016	0.389**	0.165**	0.106		
	B	3.218	1.123	0.006	-0.032	-0.123	-0.092	-0.050	-0.082		
8. Entrepreneurial intention	A	1.939	0.784	0.026	0.024	0.025	0.220**	0.222**	0.207**	0.047	
	B	3.803	0.869	0.175**	0.069	0.121	0.418**	0.411**	0.696**	-0.058	
9. Creativity	A	3.659	0.700	0.205**	0.098	-0.052	0.394**	0.502**	0.455**	0.276**	0.288**
	B	3.800	0.963	0.204**	0.089	0.137*	0.403**	0.353**	0.612**	-0.055	0.631**

*p < 0.05, **p < 0.01; A represents the Chinese sample, and B represents the African sample.

Table 3 presents the results of hierarchical regression analysis of Chinese college students sample. Table 3 shows that conscientiousness (M4, β = 0.163, *p* < 0.01) and openness (M4, β = 0.169, *p* < 0.01) had significantly positive effects on entrepreneurial intention, so the Hypothesis 1a and 2a were verified. Conscientiousness (M9, β = 0.262, *p* < 0.001) and openness (M9, β = 0.388, *p* < 0.001) were found to have significantly positive effects on creativity, so the Hypothesis 1b and 2b were verified. Boundaryless mindset (M6, β = 0.208, *p* < 0.001) was found to have a significantly positive effect on entrepreneurial intention; hypothesis 3 was verified. Boundaryless mindset (M11, β = 0.457, *p* < 0.001) was found to have a significantly positive effect on creativity; hypothesis 4 was verified. In addition, the regression analysis of entrepreneurial intention included conscientiousness, openness and boundaryless mindset. Conscientiousness (M5, β = 0.134, *p* < 0.05), openness (M5, β = 0.142, *p* < 0.05), and boundaryless mindset (M5, β = 0.125, *p* < 0.05) all had significantly positive effects on entrepreneurial intention. However, comparing Model 5 with Model 4, the regression coefficients of conscientiousness (0.134 < 0.163) and openness (0.142 < 0.169) were reduced, indicating that boundaryless mindset played a partially mediating role between conscientiousness, openness, and entrepreneurial intention; therefore, Hypotheses 5a and 6a were verified. Similarly, creativity was analyzed by regression analysis of conscientiousness, openness and boundaryless mindset. Conscientiousness (M10, β = 0.191, *p* < 0.001), openness (M10, β = 0.322, *p* < 0.001), and boundaryless mindset (M10, β = 0.304, *p* < 0.001) all had significantly positive effects on creativity. However, comparing with Model 4, the regression coefficients of conscientiousness (0.191 < 0.262) and openness (0.322 < 0.388) in Model 5 were reduced, indicating that boundaryless mindset played a partially mediating role between conscientiousness, openness, and creativity, so Hypotheses 5b and 6b were verified. Moreover, this study used Bootstrapping method and repeated sampling 5,000 times to further test the significance of indirect effects (45). The results showed that conscientiousness had significantly indirect effects on entrepreneurial intention (95% CI [0.009, 0.060]) and creativity (95% CI [0.036, 0.095]) through boundaryless mindset; openness had significantly indirect effects on entrepreneurial intention (95% CI [0.012, 0.067]) and creativity (95% CI [0.041, 0.105]) *via* boundaryless mindset.

**TABLE 3:** Hypothesis test results of Chinese samples.

Variables	Boundaryless mindset		Entrepreneurial intention					Creativity				
	**M1**	**M2**	**M3**	**M4**	**M5**	**M6**	**M7**	**M8**	**M9**	**M10**	**M11**	**M12**
Gender	-0.019	-0.048	0.027	0.004	0.010	0.031	-0.001	0.202***	0.150***	0.164***	0.210***	0.172***
Career development guidance	0.127*	0.085	0.021	-0.011	-0.021	-0.005	-0.013	0.102	0.031	0.006	0.044	0.049
Entrepreneurship courses	0.038	0.049	0.023	0.031	0.025	0.015	-0.005	-0.055	-0.039	-0.054	-0.073	-0.090
Conscientiousness		0.232***		0.163**	0.134*				0.262***	0.191***		
Openness		0.217***		0.169**	0.142*				0.388***	0.322***		
Boundaryless mindset					0.125*	0.208***	0.202***			0.304***	0.457***	0.430***
GPA							0.044					0.231***
Boundaryless mindset X GPA							0.210***					0.134**
*F*	2.063	11.286***	0.191	5.080***	5.043***	3.664**	5.014***	6.112***	31.718***	36.923***	27.778***	25.063***
*R* ^2^	0.019	0.151	0.002	0.074	0.087	0.044	0.087	0.054	0.333	0.412	0.259	0.322
△*R*^2^	0.019	0.132	0.002	0.072	0.013	0.042	0.042	0.054	0.279	0.079	0.205	0.017

*p < 0.05, **p < 0.01, ***p < 0.001, double-tailed test.

**FIGURE 2:**
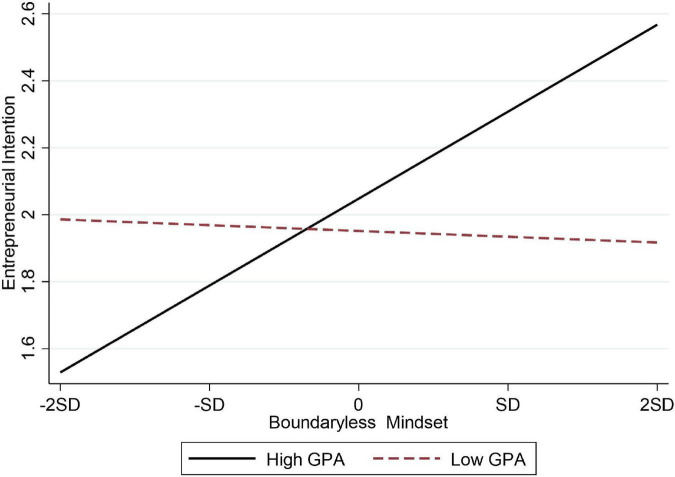
The moderating effect of GPA on the relationship between boundaryless mindset and entrepreneurial intention in Chinese samples.

**FIGURE 3:**
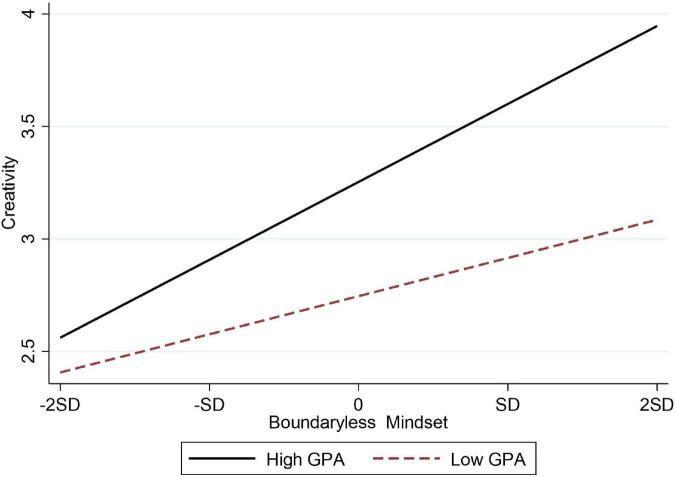
The moderating effect of GPA on the relationship between boundaryless mindset and creativity in Chinese samples.

Hypothesis 7a and 7b, respectively, assumed that GPA had a moderating effect on the relationship between boundaryless mindset and entrepreneurial intention and the relationship between boundaryless mindset and creativity. Table 3 shows that the interaction between boundaryless mindset and GPA had a significantly positive effect on entrepreneurial intention (M7, β = 0.210, *p* < 0.001) and creativity (M12, β = 0.134, *p* < 0.01). Hypothesis 7a and 7b were verified, the moderating effect diagrams were shown in Figures 2, 3, respectively.

Table 4 shows the results of hierarchical regression analysis of African college students. It shows that conscientiousness (M4, β = 0.250, *p* < 0.001) and openness (M4, β = 0.245, *p* < 0.001) had significantly positive effects on entrepreneurial intention; Hypothesis 1a and 2a were verified. Conscientiousness (M9, β = 0.275, *p* < 0.001) and openness (M9, β = 0.160, *p* < 0.05) had significantly positive effects on creativity; Hypothesis 1b and 2b were verified. Boundaryless mindset (M6, β = 0.684, *p* < 0.001) had a significantly positive effect on entrepreneurial intention; Hypothesis 3 was verified. Boundaryless mindset (M11, β = 0.593, *p* < 0.001) had a significantly positive effect on creativity; Hypothesis 4 was verified. In addition, the regression analysis of entrepreneurial intention included conscientiousness, openness and boundaryless mindset showed that boundaryless mindset (M5, β = 0.624, *p* < 0.001) had a significantly positive effect on entrepreneurial intention; however, the respective effect of conscientiousness and openness on entrepreneurial intention was no longer significant, indicating that boundaryless mindset played a complete mediating role between conscientiousness, openness and entrepreneurial intention, so Hypothesis 5a and 6a were verified. Similarly, creativity was analyzed using the regression analysis which included conscientiousness, openness and boundaryless mindset. Boundaryless mindset (M10, β = 0.539, *p* < 0.001) had a significantly positive effect on creativity, but the effect of conscientiousness and openness on creativity was no longer significant, indicating that boundaryless mindset played a complete mediating role between conscientiousness, and both openness and creativity. Hypothesis 5b and 6b were verified. In addition, this study further used Bootstrapping method repeated sampling 5,000 times to test the significance of indirect effects Preacher and Hayes (45). The results showed that conscientiousness had a significant indirect effect on entrepreneurial intention (95% CI [0.129, 0.240]) and creativity (95% CI [0.119, 0.234]) through boundaryless mindset; openness had a significant indirect effect on entrepreneurial intention (95% CI [0.124, 0.234]) and creativity (95% CI [0.121, 0.235]) through boundaryless mindset.

**TABLE 4:** Hypothesis test results of African samples.

Variables	Boundaryless mindset		Entrepreneurial intention					Creativity				
	**M1**	**M2**	**M3**	**M4**	**M5**	**M6**	**M7**	**M8**	**M9**	**M10**	**M11**	**M12**
Gender	0.155*	0.081	0.167*	0.102	0.051	0.061	0.072	0.197**	0.137*	0.093	0.105*	0.110*
Career development guidance	-0.029	-0.033	0.048	0.043	0.064	0.067	0.068	0.068	0.063	0.081	0.085	0.085
Entrepreneurship courses	0.060	-0.081	0.082	-0.041	0.010	0.041	0.033	0.087	-0.021	0.022	0.051	0.047
Conscientiousness		0.266***		0.250***	0.084				0.275***	0.132		
Openness		0.302***		0.245***	0.056				0.160*	-0.003		
Boundaryless mindset					0.624***	0.684***	0.687***			0.539***	0.593***	0.594***
GPA							0.016					0.007
Boundaryless mindset X GPA							-0.119*					-0.056
*F*	2.425	16.771***	3.462*	13.163***	39.640***	57.384***	40.096***	4.815**	11.436***	26.882***	38.633***	25.862***
*R* ^2^	0.030	0.265	0.043	0.221	0.507	0.496	0.510	0.058	0.198	0.411	0.399	0.402
△*R*^2^	0.030	0.235	0.043	0.178	0.286	0.454	0.014	0.058	0.140	0.213	0.341	0.003

*p < 0.05, **p < 0.01, ***p < 0.001, double-tailed test.

**FIGURE 4:**
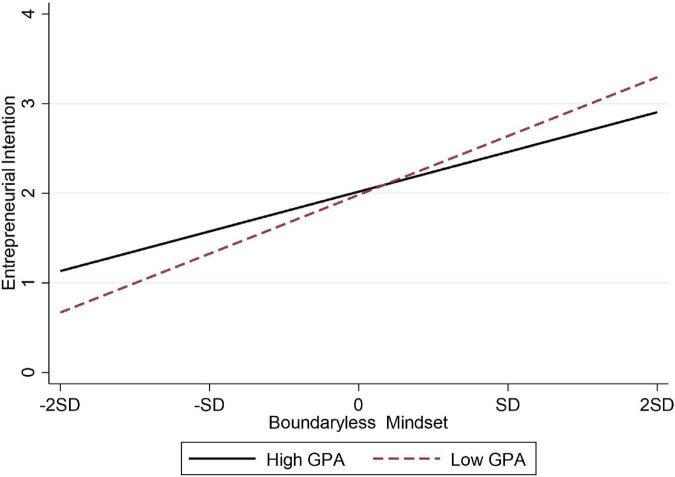
The moderating effect of GPA on the relationship between boundaryless mindset and entrepreneurial intention in African samples.

Hypothesis 7a and 7b, respectively, assumed that GPA has a moderating effect on the relationship between boundaryless mindset and entrepreneurial intention and the relationship between boundaryless mindset and creativity. As seen from Table 4, the interaction between boundaryless mindset and GPA had a significant negative impact on entrepreneurial intention (M7, β = 0.210, *p* < 0.001), so Hypothesis 7a was verified; the moderating effect diagram was shown in Figure 4. However, interaction had no significant effect on creativity, so Hypothesis 7b was not supported.

## Discussion

This study chose Chinese and African college students as research objects, and empirically tested the effect of conscientiousness and open personality traits on entrepreneurial intention and creativity from the perspective of boundaryless mindset under the context of Industry 4.0/5.0. The results showed that: (1) In the samples of Chinese and African college students, both conscientiousness and openness had significantly positive effects on entrepreneurial intention and creativity, respectively. (2) Boundaryless mindset served as a mediator in the relationship between conscientiousness, openness and entrepreneurial intention and creativity of college students. In the Chinese and African college students sample, this mediating effects are slightly different. In the Chinese sample, boundaryless mindset played a partial mediating role; in the African sample, boundaryless mindset played a complete mediating role. (3) The moderating effects of GPA were quite different in Chinese and African samples. In the Chinese sample, GPA strengthened the effect of boundaryless mindset on entrepreneurial intention and creativity. In African samples, GPA weakened the influence of boundaryless mindset on entrepreneurial intention and had no significant influence on creativity. This may mainly be due to the differences in economic development and educational levels between China and Africa. Since China’s economic reform and opening-up, international trading and the economic development has been increasing steadily, and entrepreneurship has been encouraged with many opportunities. At the same time, many college students in China are the only child in their family. These families have relatively less financial burden than families with multiple children, so more money and resources can be saved up and invested in the child’s personal development, and even entrepreneurial aspirations. Previous studies have pointed out that more resources and financial support can help promote individuals’ creativity and entrepreneurial intention (46, 47). Furthermore, high GPA means a college student is better at learning and handling information. Studies have shown that learning capability and information disposition capability can positively influence individuals’ creativity and the ability to identify entrepreneurial opportunities (3, 48). Under the influence of multiple factors, Chinese college students are more likely to pursuit entrepreneurial opportunities or innovative activities. In contrast, many African countries have relatively poorly developed economies which offer less entrepreneurial opportunities and resources. Some regions are even deprived of the most basic living needs. Students with excellent academic performances are usually favored by stable, high-paying jobs from established firms. These students facing the depressed situation and high economic uncertainty may prefer these firms over entrepreneurship since good jobs can also ease the financial burden in their families. In addition, in African samples, GPA did not significantly strengthen the effect of boundaryless mindset on creativity. This may be due to the relatively poor economic development in Africa, resulting in a poorer quality of higher education. It is also difficult to ascertain if the GPA of African college students truly reflected their comprehensive learning ability and quality. This provides a potential explanation to why it had no obvious influence on the relationship between boundaryless mindset and creativity in our study.

The theoretical contribution of this study is mainly presented in two aspects. First of all, different from the traditional bounded career, this study focused on the emerging boundaryless career from the perspective of individuals’ boundaryless career attitude. It explored the effect of college students’ conscientiousness and open personality traits on entrepreneurial intention and creativity, and expanded the theoretical research on the career development of modern college students in the context of Industry 4.0/5.0. Secondly, this study took college students in China and Africa as research objects to study the relationship between personality traits, boundaryless mindset, academic performance (GPA), entrepreneurial intention and creativity. This is an empirical study of college students trained by different level of higher education quality under different cultural and economic backgrounds, which expanded the theoretical research on the influencing factors of entrepreneurial intention and creativity of college students from different background situations.

This study also has important implications on how higher education can foster students to adapt to the employment environment under the background of Industry 4.0/5.0. Firstly, Industry 4.0/5.0 has brought us into a digitized, intelligent and networking age and employment has become more diverse and flexible, the path and form of individual career development is more diversified. Younger generations increasingly favor the boundaryless career development mode over the traditional boundary career development mode (49). Both Chinese or African countries are developing countries, and they need to continuously develop more cutting-edge technology or change industry forms in the era of Industry 4.0/5.0, which requires more creative and entrepreneurial organizations or employees. Higher education should pay more attention at developing college students’ boundaryless mindset to help them adapt to future employment environment, further increasing their creativity and entrepreneurial intention and even better work performance. For example, colleges should provide more courses and opportunities for cross-disciplinary studies, in addition to the required courses, for students to learn knowledge and skills from different professional fields, interact with students from different majors, and improve their comprehensive capabilities. Secondly, during the process of encouraging and guiding college students’ innovation and entrepreneurship, it is necessary to focus on students’ academic performances and the shaping of their personalities. We need to pay close attention both to the cultivation of open personality traits, as well as their responsible and conscientious personality traits, which further promotes students’ creativity and entrepreneurial intention. Modern higher education has paid more attention on its influences over students’ personality, and many scholars believed that is very important to talent cultivation (50). This research validated that shaping personality traits can improve students’ capability and even their future performances and achievement. Thirdly, from the moderating effects of GPA, we have discovered that GPA levels from different countries can reflect different capability levels. China has a relatively higher level of education quality that GPA can reflect students’ comprehensive capabilities more accurately, but not in Africa. Therefore, international society should send more experts and teachers to Africa to launch aid projects, provide more training to teachers in African higher education and share educational experiences to implement better curriculum design and educational management. These efforts may improve the quality and effectiveness of the evaluation of college students’ academic performance, thus to better reflect African college students’ comprehensive learning capability and quality *via* GPA.

Despite these important implications from this comparative study, there are some limitations that should be addressed in future research. First, this was a cross-sectional study in which the data was collected from a common source. Although the data deviation test showed that the results were in an acceptable range, it might not completely reflect the dynamic causal relationship between variables. Future research can use time series data to collect data of different variables in stages, providing more powerful data support for the causal relationship between variables. Secondly, this study had constraints on sample size and representativeness. The Chinese samples came from a single college in Shanghai, while the African samples are mainly from universities in the Democratic Republic of the Congo. Expanding the number and regional sources of samples would greatly improve the representativeness of this research topic.

## Data availability statement

The raw data supporting the conclusions of this article will be made available by the authors, without undue reservation.

## Ethics statement

The studies involving human participants were reviewed and approved by Ethics Committee of Shanghai Jiao Tong University. The patients/participants provided their written informed consent to participate in this study.

## Author contributions

MX conceived the structure of the manuscript, wrote the manuscript, collected the data, analyzed the data, and approved the submitted version.
